# Apospory and Diplospory in Diploid *Boechera* (Brassicaceae) May Facilitate Speciation by Recombination-Driven Apomixis-to-Sex Reversals

**DOI:** 10.3389/fpls.2019.00724

**Published:** 2019-05-31

**Authors:** John G. Carman, Mayelyn Mateo de Arias, Lei Gao, Xinghua Zhao, Becky M. Kowallis, David A. Sherwood, Manoj K. Srivastava, Krishna K. Dwivedi, Bo J. Price, Landon Watts, Michael D. Windham

**Affiliations:** ^1^Plants, Soils and Climate Department, Utah State University, Logan, UT, United States; ^2^Instituto Tecnológico de Santo Domingo, Santo Domingo, Dominican Republic; ^3^College of Desert Control Science and Engineering, Inner Mongolia Agricultural University, Hohhot, China; ^4^Caisson Laboratories, Inc., Smithfield, UT, United States; ^5^Crop Improvement Division, Indian Grassland and Fodder Research Institute, Jhansi, India; ^6^Department of Biology, Duke University, Durham, NC, United States

**Keywords:** apomeiosis, apomixis, apomixis-to-sex reversion, apospory, recombination driven diploidization, *Boechera* (Brassicaceae), diplospory, reticulate evolution

## Abstract

Apomixis (asexual seed formation) in angiosperms occurs either sporophytically, through adventitious embryony, or gametophytically, where an unreduced female gametophyte (embryo sac) forms and produces an unreduced egg that develops into an embryo parthenogenetically. Multiple types of gametophytic apomixis occur, and these are differentiated based on where and when the unreduced gametophyte forms, a process referred to as apomeiosis. Apomeiotic gametophytes form directly from ameiotic megasporocytes, as in Antennaria-type diplospory, from unreduced spores derived from 1st division meiotic restitutions, as in Taraxacum-type diplospory, or from cells of the ovule wall, as in Hieracium-type apospory. Multiple types of apomeiosis occasionally occur in the same plant, which suggests that the different types occur in response to temporal and/or spatial shifts in termination of sexual processes and onset timing of apomeiosis processes. To better understand the origins and evolutionary implications of apomixis in *Boechera* (Brassicaceae), we determined apomeiosis type for 64 accessions representing 44 taxonomic units. Plants expressing apospory and diplospory were equally common, and these generally produced reduced and unreduced pollen, respectively. Apospory and diplospory occurred simultaneously in individual plants of seven taxa. In *Boechera*, apomixis perpetuates otherwise sterile or semisterile interspecific hybrids (allodiploids) through multiple generations. Accordingly, ample time, in these multigenerational clones, is available for rare meioses to produce haploid, intergenomically recombined male and female gametes. The fusion of such gametes could then produce segmentally autoploidized progeny. If sex re-emerges among such progeny, then new and genomically unique sexual species could evolve. Herein, we present evidence that such apomixis-facilitated speciation is occurring in *Boechera*, and we hypothesize that it might also be occurring in facultatively apomictic allodiploids of other angiospermous taxa.

## Introduction

The genus *Boechera* (Brassicaceae) evolved about 2.5 Myr ago (Mandakova et al., 2015) and is closely related to *Arabidopsis* (Bailey et al., 2006; Rushworth et al., 2011). It encompasses c. 83 primarily inbreeding sexual diploid taxa (Li et al., 2017), many of which have relatively narrow geographic ranges. *Boechera* also includes hundreds of genomically distinct diploid, triploid and tetraploid hybrids that are partially to fully sterile sexually. These hybrids produce most of their seeds through apomixis (without meiotic recombination, chromosome reduction or fertilization), but sexually derived seeds are also occasionally produced (Aliyu et al., 2010). This dual capacity, to produce seeds sexually and apomictically, is called facultative apomixis, and it is characteristic of most if not all angiospermous apomicts (Asker and Jerling, 1992).

Most *Boechera* taxa belong to a well-supported western North American clade (Alexander et al., 2013), the distribution of which extends from northern Mexico to the Arctic with outlying populations (mostly apomictic and polyploid) in Greenland and around the Great Lakes and the St. Lawrence River. Another clade of nine taxa, previously assigned to the genus *Borodinia* (Alexander et al., 2013), is here included in *Boechera* due to the recent discovery of inter-clade hybridization (Windham et al., field observations). The latter are distinctive in being sparsely pubescent and restricted to forested regions of eastern North America and the Russian Far East. *Boechera* s.l. is, by far, the largest genus of tribe Boechereae, a morphologically disparate group that includes seven other genera whose phylogenetic affinity only became apparent through recent chromosomal and molecular analyses. Indeed, the primary defining characteristic of Boechereae consists of a reduction in chromosome base number from *n* = 8 to *n* = 7 (Al-Shehbaz, 2003). Evidence suggests that the *n* = 8 Boechereae ancestor entered North America from Asia about 5 Mya via the Bering land bridge. The chromosome base number reduction likely occurred thereafter by multiple translocations (Mandakova et al., 2015).

Though predominantly autogamous (self-pollinating), interspecific hybrids (allodiploids) involving sexual *Boechera* diploids, as well as their introgression products, are frequently encountered in nature (Kantama et al., 2007; Beck et al., 2012; Aliyu et al., 2013; Alexander et al., 2015; Li et al., 2017). These are generally apomictic and display broad ecological competencies (Windham and Al-Shehbaz, 2006; Alexander et al., 2015; Windham et al., 2015; Shah et al., 2016). Because of introgression, apomictic *Boechera* are often confused with sexual diploids, the habitats of which are generally much more difficult to locate. As with many agamic complexes (Asker and Jerling, 1992; Bayer, 1997), this situation complicates *Boechera* taxonomy (Li et al., 2017).

While apparently common in *Boechera*, apomixis arising in allodiploid hybrids, which form between two sexual diploid species, is rare in other angiosperms (Carman, 1997). In this respect, many *Boechera* apomicts also produce unreduced (2*n*) pollen, which also is generally uncommon among other angiospermous apomicts (Asker and Jerling, 1992). In *Boechera*, 2*n* sperm of apomictic diploids can fertilize 1*n* eggs of co-occurring sexual taxa to produce new and genomically unique triploid apomicts (Bocher, 1951; Alexander et al., 2015; Li et al., 2017). Apomictic *Boechera* tetraploids also arise in this manner, but these are less common (Schranz et al., 2005; Aliyu et al., 2010).

Frequent hybridization with or without homoeologous recombination (Kantama et al., 2007) explains the proliferation of apomictic alloploid *Boechera* (Beck et al., 2012; Windham et al., 2015; Li et al., 2017), but how the sexual diploids originate is less obvious. The traditional view is that they arise by range expansion and speciation along ecological gradients (Alexander et al., 2015; Li et al., 2017). However, the slow pace of such speciation is inconsistent with the large numbers of rare sexual diploids described for this youthful genus. Here we provide a cytological and theoretical framework that addresses this question.

Apomixis is verifiable by single seed flow cytometry, which measures embryo-to-endosperm genome ratios, e.g., 2C:3C seeds (diploid embryo and triploid endosperm) are sexual, but 2C:5C or 2C:6C are apomictic (Matzk et al., 2000; Aliyu et al., 2010). However, “types” of apomixis must be determined cytologically (Asker and Jerling, 1992; Hand and Koltunow, 2014). Apomixis is gametophytic in *Boechera*, which means ovules produce 2*n* female gametophytes (embryo sacs), which in turn produce parthenogenetic eggs.

The pioneering study of female meiosis (megasporogenesis) and female gametophyte formation in *Boechera* (Bocher, 1951) was motivated by observations of 2*n* pollen formation. This, plus two subsequent studies of 2*n* pollen forming *Boechera* (Naumova et al., 2001; Taskin et al., 2004), revealed meiotic first division restitutions that produced dyads of 2*n* spores in ovules and anthers. On the male side, both spores formed 2*n* pollen. On the female side, one 2*n* spore degenerated and the other developed into a 2*n* gametophyte (Taraxacum-type diplospory). This limited embryological sampling led to an incorrect notion that 2*n* and 1*n* pollen in *Boechera* are diagnostic of apomixis and sex, respectively (Roy, 1995; Windham and Al-Shehbaz, 2006). More thorough sampling in recent years has provided a clearer picture of apomixis development in *Boechera* (Carman, 2007; Carman et al., 2015). Certain accessions of *Boechera microphylla* (*B. imnahaensis* × *yellowstonensis*) were found to be highly apomictic despite apparently normal male and female meioses (Mateo de Arias, 2015). In these plants, functional pollen grains form from all four 1*n* microspores. However, on the female side, all meiotically produced spores generally degenerate, and a 2*n* gametophyte forms adventitiously from a nucellar cell of the ovule wall (Hieracium-type apospory).

To better understand the unusual pervasiveness and origins of multiple apomixis types in *Boechera*, we expanded our taxonomic sampling to 64 accessions representing 44 operational taxonomic units (OTUs). Our sampling includes sexual and apomictic taxa that span the *Boechera* phylogeny (Alexander et al., 2013), and it represents a mix of taxa traditionally treated as species as well as recently discovered but as yet unpublished entities. Hereafter, published names are used for the diploid sexual species. However, the apomictic hybrids are identified by genome composition as found in the *Boechera* Microsatellite Website (BMW) http://sites.biology.duke.edu/windhamlab/ (Li et al., 2017). We show that both apospory (normal male and female meioses with female sexual development failing thereafter) and diplospory (first division restitution male and female meioses) occur frequently in *Boechera* and are widely dispersed across the genus. Based on these findings, we provide a possible explanation for the origins of rare and allelically poor sexual endemics that are often encountered in habitats otherwise populated by allelically complex apomicts.

## Materials and Methods

### Plant Materials

Cytological analyses were performed using floral buds taken from plants growing in native habitats, plants transplanted from native habitats, or plants grown from seeds (Supplementary Table S1). Seeds were placed on moist filter paper, stratified at 4°C for 21 days, and planted. Potted seedlings or transplants were grown in 600 mL cone-shaped (68 mm diameter × 255 mm tall) pots or 350 mL square (85 mm wide × 95 mm tall) pots that contained Sunshine Mix #1 potting soil (Sun Gro Horticulture Canada Ltd., Vancouver, BC, Canada). Vernalization was accomplished by cold incubation (4°C) for 10–12 weeks with minimal lighting (8/16 day/night photoperiod). Vernalized plants were transferred to controlled-environment greenhouses or growth chambers that maintained a 16/8 h day/night photoperiod using supplemental light provided by a combination of cool white florescent bulbs, incandescent bulbs, and high-pressure sodium-vapor lamps. These provided a minimum photosynthetic photon flux of 400 μmol m^-2^ s^-1^ at the tops of the canopies. Day/night temperatures were maintained at 22/16°C, and plants were watered regularly with a dilute solution (250 mg L^-1^) of Peters Professional 20:20:20 fertilizer (Scotts, Maryville, OH, United States).

### Cytological Analyses

Clusters of floral buds at pre-anthesis stages were fixed in formalin acetic acid alcohol (FAA) or Farmer’s 3:1 fixative (ethanol acetic acid) for 48 h. The buds were then cleared in 2:1 benzyl benzoate dibutyl phthalate (BBDP) (Crane and Carman, 1987) as follows: 70% EtOH, 30 min; 95% EtOH, 4 h (2×); 2:1 95% EtOH BBDP, 2 h; 1:2 95% EtOH BBDP, 4 h; 100% BBDP, 4 h; and 100% BBDP overnight (kept until analyzed). Pistils were then dissected from the floral buds. Pistil lengths, measured from the base of the pedicel to the top of the stigma (±0.05 mm), were then obtained using a dissection microscope, and the pistils were mounted on slides with a minimal amount of 2:1 BBDP clearing solution. Development was studied using an Olympus (Center Valley, PA, United States) BX53 microscope equipped with differential interference contrast (DIC) optics, an Olympus DP74 digital camera, and Olympus cellSens Dimension Version 1 software.

The following ovule stages were tabulated: (i) pre-meiotic megaspore mother cell (MMC), (ii) meiotic or diplosporous dyad, (iii) sexual tetrad of megaspores, (iv) enlarged functional megaspore with three degenerating megaspores (sexually derived), (v) early-stage 1 or 2-nucleate gametophyte with three degenerating megaspores, (vi) enlarged functional megaspore with one degenerating megaspore (Taraxacum-type diplospory), (vii) early-stage 1 or 2-nucleate gametophyte with one degenerating megaspore (Taraxacum-type diplospory), (viii) early-stage 1 or 2-nucleate gametophyte with no degenerating megaspores (Antennaria-type diplospory), (ix) presence of one or more enlarged non-vacuolate nucellar cells (aposporous initials, AI) that equaled or surpassed the size of the meiocyte (meiotically active MMC, dyad or early-staged tetrad), and (x) enlarged nucellar cell(s) with one or more distinct vacuoles and 1–2 nuclei (early-stage aposporous gametophytes, AES). Because of uncertainties in origin, gametophytes (sexual, diplosporous or aposporous) were not recorded beyond the 2-nucleate stage. Pistil lengths and developmental stages of the majority of scorable ovules in each pistil were recorded.

### Flow Cytometry

**FIGURE 1:**
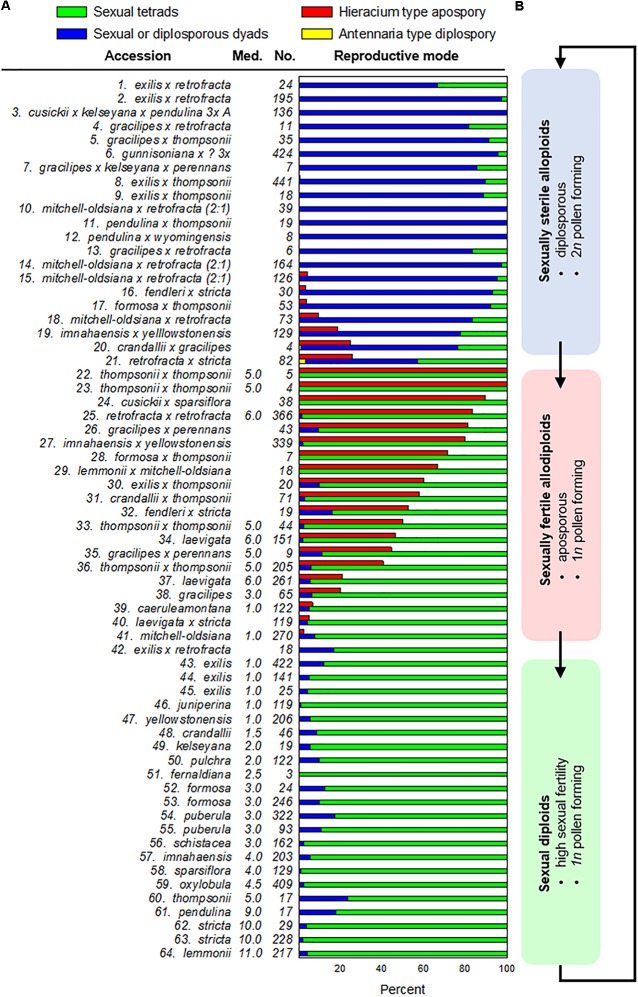
Meiosis and apomeiosis in 64 *Boechera* accessions (organized by reproductive mode). **(A)** Frequencies by taxon of ovules exhibiting sexual tetrads, sexual or Taraxacum-type diplosporous dyads, Hieracium-type aposporous gametophytes, or Antennaria-type diplosporous gametophytes (accession numbers correspond to those in Supplementary Table S1). Tetrad, dyad, and Antennaria-type diplosporous gametophyte frequencies per accession sum to 100%. Aposporous gametophyte frequencies are listed separately (red bars). These develop adventitiously while meiotic tetrads form and degenerate. Median (Med.) numbers of SSR alleles observed among homozygous samples of each sexual accession (Supplementary Table S2) are shown (Med.) as are numbers (No.) of correctly staged ovules analyzed per accession. **(B)** A hypothesis of evolutionary cycling between hybridization induced apomixis and apomixis facilitated reticulate evolution of new sexual species. Blue box: taxa with mostly 2*n* pollen with some reduced and shrunken pollen, which suggests meiotic anomalies due to recent interspecific hybridization but with a transition from diplospory to apospory occurring in some taxa possibly due to early genome diploidization events associated with infrequent selfing (15–21); red box: taxa with mostly fertile reduced pollen coupled with apospory and sexual tetrad formation and degeneration, which suggests more extensive genome diploidization with a gradual restoration of sexual fertility; green box: sexually fertile anthers and pistils with no cytological evidence of apomeiosis.

Relative levels of nuclear DNA in embryo and endosperm cells of single seeds of *B. stricta* (Figure 1, 63), *B. exilis* ×*thompsonii* (Figure 1, 8), *B. imnahaensis* × *yellowstonensis* (Figure 1, 27) and *B. cf. gunnisoniana* 3× (Figure 1, 6) were determined. Nuclei of immature and mature seeds were isolated using a mortar, pestle and a few drops of DAPI (4,6-diamidino-2-phenylindole) containing Partec (Partec North America, Inc., Swedesboro, NJ, United States) buffer (CyStain UV Precise P). Pestles were used to lightly crush the seeds. Fragments were exposed to buffer for several minutes. The nuclei-containing solutions were then filtered through 30 μm nylon filters into 1.2 mL tubes. Nuclear fluorescence was determined using a Partec PA flow cytometer. Seeds with a 2C:3C embryo endosperm ratio were recorded as sexual, while seeds with 2C:5C, 2C:6C, 2C:7C, or 3C:9C ratios were recorded as apomictic (Aliyu et al., 2010).

### Allelic Diversity and Geographic Distributions of Sexual Diploids

For sexual diploids, taxonomic names, specimen numbers, locations of origin, and allele lengths for 13 single-locus microsatellite (simple sequence repeat, SSR) loci (A1, BF3, B6, B9, B11, BF15, B18, B20, B266, C8, E9, I3, and I14) were downloaded from the BMW. To minimize inclusion of apomicts (mistakenly collected as sexual diploids), specimens were excluded if they were heterozygous for any of the 13 loci, yielded data for less than six of the 13 loci, or represented taxa with less than six homozygous specimens. Taxa meeting these criteria were then ranked based on median and mean numbers of alleles per locus (population level allelic polymorphism). This variability was used to identify putative sexual or apomictic ancestors.

## Results

Clearing and mounting of whole pistils using BBDP (Crane and Carman, 1987) was efficient for high throughput analysis of megasporogenesis and gametophyte formation. Each pistil contained, depending on species, from 40 to 200 ovules (Al-Shehbaz et al., 2010). When pistils were mounted horizontally (c. 16 per slide), 20–30% of their ovules were in sagittal orientation, which permitted efficient analyses of MMC origins as well as details of dyad, tetrad and early gametophyte formation.

### Diplospory and Apospory Are Common in *Boechera*

**FIGURE 2:**
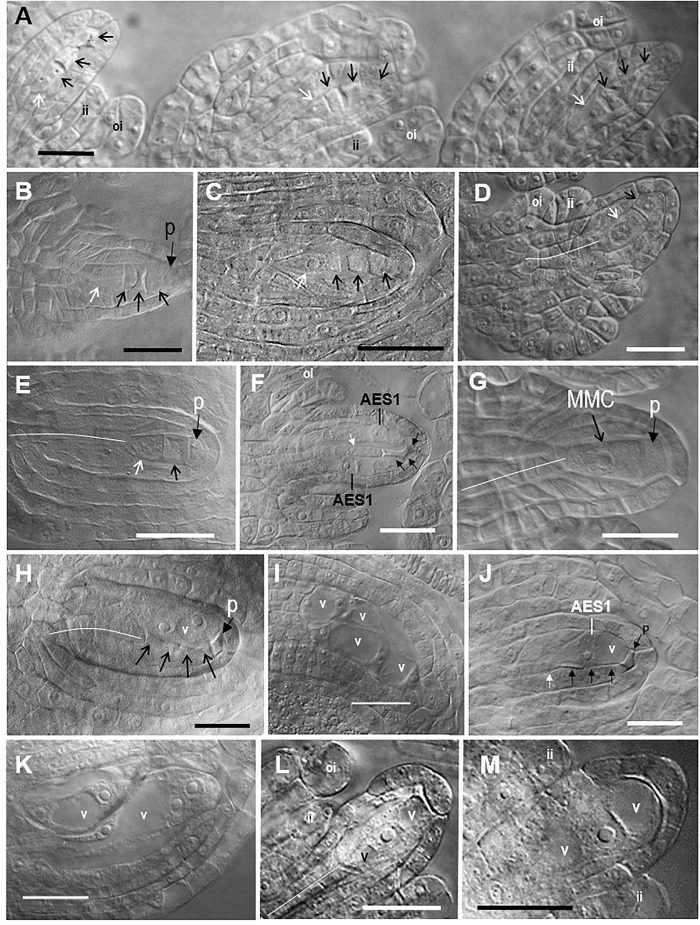
Representative images of sexual megasporogenesis and Taraxacum-type diplosporous, Hieracium-type aposporous, and Antennaria-type diplosporous gametophyte (ES) development in *Boechera*. **(A)** Row of three adjacent sexual tetrads in a *B. stricta* ovule (Figure 1, 63), **(B)** sexual tetrad in a *B. yellowstonensis* ovule (Figure 1, 47), **(C)** sexual tetrad in a *B. exilis* ovule (Figure 1, 43), **(D)** unreduced Taraxacum-type dyad in a *B. exilis* × *thompsonii* ovule (Figure 1, 8), **(E)** unreduced Taraxacum-type dyad in a *B. crandallii* × *gracilipes* ovule (Figure 1, 20), **(F)** two unreduced 1-nucleate Hieracium-type aposporous ESs with a degenerating tetrad in a *B. retrofracta* ×*stricta* ovule (Figure 1, 21), **(G)** sexual megaspore mother cell (MMC) with a parietal cell in a *B. yellowstonensis* ovule (Figure 1, 47), **(H)** unreduced 2-nucleate Hieracium-type aposporous ES with a degenerating sexual tetrad in a *B. cusickii* ×*sparsiflora* ovule (Figure 1, 24), **(I)** four unreduced 2-nucleate Hieracium-type aposporous ES in a *B. imnahaensis* × *yellowstonensis* ovule (Figure 1, 19), **(J)** unreduced 1-nucleate Hieracium-type aposporous ES with a degenerating tetrad in a *B. retrofracta* ×*retrofracta* ovule (Figure 1, 25); **(K)** an unreduced 2-nucleate and an unreduced 4-nucleate aposporous gametophyte in a *B. crandallii* × *thompsonii* ovule (Figure 1, 31), **(L,M)** unreduced 1-nucleate Antennaria-type diplosporous ES forming directly from the MMC in a *B. retrofracta* × *stricta* ovule (Figure 1, 21). Black arrows, degenerating megaspores; white arrows, surviving megaspores; narrow white lines, central column of nucellar cells, which gave rise to the archesporial cell; ai, aposporous initial cell; ii, inner integument; oi, outer integument; p, parietal cell; v, vacuole; bars, 20 μm.

In most sexual angiosperms, the mature female gametophyte is a seven celled (eight nucleate) structure (Polygonum type) that forms from a genetically reduced megaspore of a meiotic tetrad (Johri et al., 1992). Accordingly, the consistent observation of the following four phenomena was taken as strong evidence for near-obligate to obligate sexual reproduction: (i) meiotic tetrad formation, (ii) absence of aposporous gametophytes at the meiotic tetrad stage, (iii) absence of vacuolate enlargement or endomitotic activity in a dyad obtained from a MMC, and (iv) vacuolate enlargement of the surviving megaspore of a meiotic tetrad coupled with endomitotic activity. These criteria were consistently observed in the ovules of 23 of the 64 accessions analyzed (Figure 1A, 42–64). In these accessions, the tetrad to functional megaspore stage lasted c. 2 d, which permitted tetrads to accumulate in rows along the placentae (Figure 2A–C). Additional photomicrographs diagnostic of sexual ovule development (meiotic tetrads and vacuolate 1- to 2-nucleate gametophytes forming from surviving megaspores of meiotic tetrads) are shown in Supplementary Figures S1A–P for eight of the sexual taxa identified herein.

Ovules from 21 of the 64 accessions analyzed (Supplementary Table S1, 16 OTUs) generally underwent Taraxacum-type diplospory (Figure 1A, 1–21). Here, a first division meiotic restitution occurred, which was followed by a mitotic-like second division to produce a dyad of 2*n* megaspores (Figure 2D,E). The 2*n* gametophyte then developed from the chalazal most spore (Figure 2D), and the micropylar-most spore degenerated. The dyad stage terminated Taraxacum type diplosporous megasporogenesis and, as with sexual megasporogenesis (resulting in tetrads), a pause in development preceded gametophyte formation. These pauses allowed diplosporous dyads to accumulate in rows, like sexual tetrads, along the ovary placentae (compare Figure 2A with Supplementary Figure S2A). Additional photomicrographs diagnostic of Taraxacum-type diplosporous ovule development (vacuolate 1- to 2-nucleate unreduced gametophytes forming from surviving megaspores of unreduced apomeiotic dyads) are shown in Supplementary Figures S2A–F for six of the Taraxacum-type diplosporous taxa identified herein. Also shown are unreduced microspore dyads (Supplementary Figure S2G), which are commonly produced in the anthers of diplosporous *Boechera* (Bocher, 1951; Naumova et al., 2001).

Aposporous gametophytes generally replaced all four megaspores of meiotic tetrads in 27 of 64 accessions, which represented 19 OTUs (Figure 1A, 15–41, 2H–K). Photomicrographs diagnostic of apospory (degenerating meiotic tetrads being replaced by vacuolate 1- to 2-nucleate aposporous gametophytes) are shown in Supplementary Figures S2H–O for seven aposporous taxa. Because we scored reproduction from the dyad to 2-nucleate gametophyte stages, our apospory frequencies are probably underestimates. This is because some ovules scored as sexual in the dyad to early tetrad stages would have likely produced aposporous gametophytes had they been fixed at a later date. Apospory and diplospory occurred together in seven of the 27 aposporous accessions (Figure 1A, 2F).

Antennaria-type diplospory (mitotic diplospory or gonial apospory) occurred rarely (Figure 1A). When it did occur, it began early in ovule development while inner and outer integuments were initiating (Figure 2L,M). To our knowledge, this is the first report of Antennaria-type diplospory in *Boechera*. Aposporous gametophytes also began to form during early integument development (Figure 2F,J and Supplementary Figures S2M,N). In contrast, gametophyte formation from functional megaspores of sexual tetrads (Figure 2A and Supplementary Figures S1D,F) and Taraxacum-type diplosporous dyads (Figure 2D and Supplementary Figures S2C–E) generally occurred as integuments were enclosing the nucellus.

In angiosperms, the nucellus develops by periclinal divisions of subepidermal cells of the funiculus, and the cells of the epidermis divide anticlinally to accommodate this nucellar enlargement. The periclinal divisions produce columns of nucellar cells. The central column extends from the middle of the chalaza to the distil most position at the micropylar epidermis (see narrow white lines, Figure 2D,E,G,H,L and Supplementary Figures S1I,J, S2B,D,K,L,N). The distil cell of the central column enlarges to produce the archesporial cell. In some angiosperms, enlarging archesporial cells divide mitotically to produce a parietal cell that separates the archesporial cell from the nucellar epidermis (Johri et al., 1992). In our sexual and apomictic taxa, parietal cells formed in 10–20% of the ovules, and they were observed from the MMC stage until they degenerated during early gametophyte formation (Figure 2B,E,G,H and Supplementary Figures S1C,D,G,L,O, S2C,I,K–M). Histochemical evidence suggests that abnormal meioses can also produce parietal-like cells in *Boechera* (Rojek et al., 2018).

**FIGURE 3:**
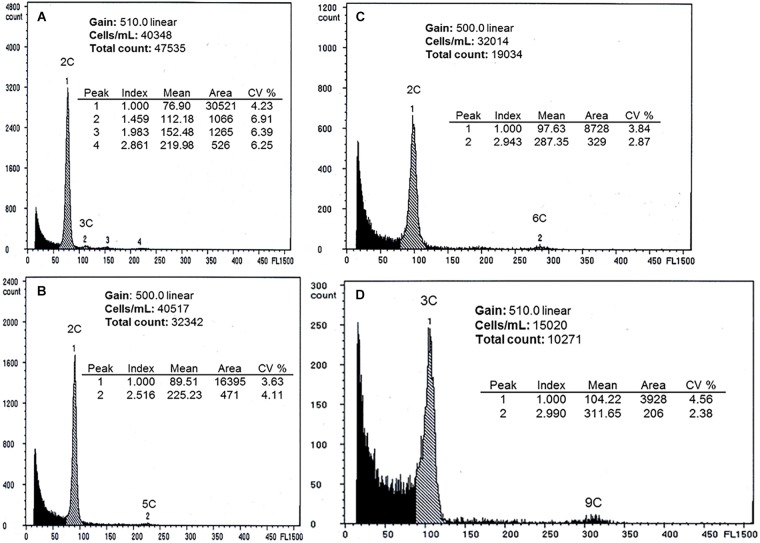
Results of single-seed flow cytometry. **(A)** sexual seed of *B. stricta* (Figure 1, 63), 2C embryo peak, 3C endosperm peak, **(B)** apomictic seed of aposporous *B. imnahaensis* ×*yellowstonensis* (Figure 1, 27), 2C embryo peak, 5C endosperm peak (fusion of two unreduced polar nuclei with a reduced pollen nucleus), **(C)** apomictic seed of *B. exilis* ×*thompsonii* (Figure 1, 8), 2C embryo peak, 6C endosperm peak (fusion of two unreduced polar nuclei with an unreduced pollen nucleus), and **(D)** apomictic seed of triploid *B. gunnisoniana* × ? 3× (Figure 1, 6), 3C embryo peak, 9C endosperm peak (fusion of two unreduced polar nuclei with an unreduced pollen nucleus). The small peaks corresponding to 4C (visible in panels **A,C**) may be embryonal nuclei in the G2 cell cycle phase or pairs of embryonal nuclei stuck together.

Four taxa were studied by flow cytometry. Seeds from diploid aposporous *B. imnahaensis* ×*yellowstonensis* generally produced peaks consistent with unreduced gametophyte central cells (4C, from the fusion of two 2*n* polar nuclei) being fertilized by 1*n* sperm nuclei (1C). Of 47 seeds successfully tested, 44 exhibited the expected 2C:5C ratio, consistent with reduced pollen formation, two exhibited a sexual 2C:3C ratio, and one exhibited a 2C:7C ratio, which likely reflects 4C central cell fertilization by a 3C sperm from adjacently growing diplosporous triploid *B. c.f. gunnisoniana* 3×. This 96% apomictic seed set confirms our suspicion that aposporous gametophyte frequencies (80% for this taxon; Figure 1A, 27) underestimate apomixis penetrance. All seven peak-producing seeds of *B. cf. gunnisoniana* 3× exhibited the expected 3C:9C ratio for this diplosporous triploid. Of 48 peak-producing seeds of diplosporous *B. exilis* ×*thompsonii*, 45 produced a 2C:6C ratio, consistent with unreduced pollen formation, two produced a sexual 2C:3C ratio, and one produced a 2C:7C ratio. The latter again suggests fertilization by the adjacently growing *B. cf. gunnisoniana* 3×. The 24 *B. exilis* ×*retrofracta* and the two *B. retrofracta* ×*stricta* peak-producing seeds produced the expected diplosporous 2C:6C ratio. Likewise, all 12 seeds from the sexual *B. stricta* produced the expected sexual 2C:3C ratio (Figure 3).

### Evidence for Homoeologous-Recombination-Driven Reticulation

**FIGURE 4:**
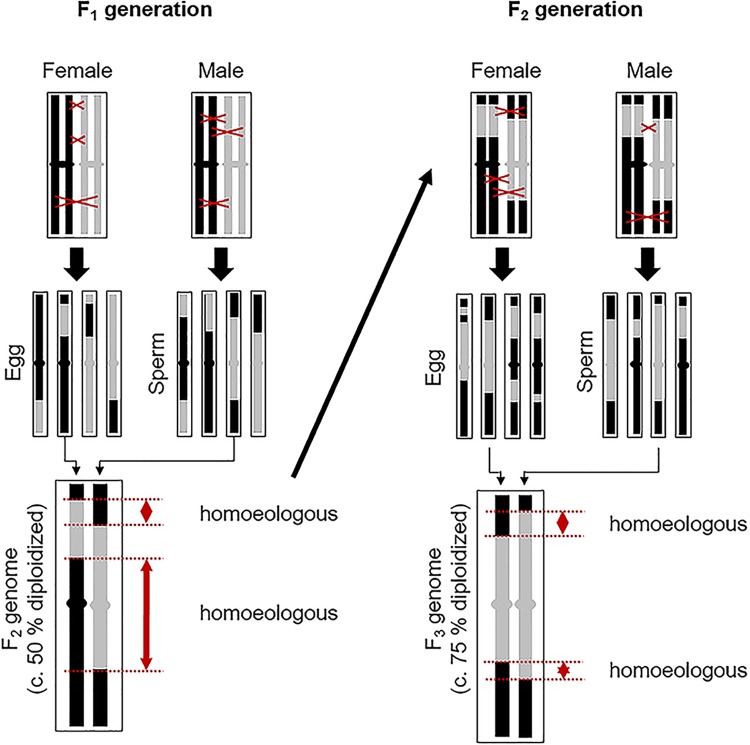
Process whereby a facultatively aposporous allodiploid may produce new genomically unique sexual species consisting of alternating sections of homologous chromosomal regions from its homoeologous parental genomes. One homoeologous chromosome pair is represented. Black and gray chromosome regions are homoeologous. On average, remaining homoeology is decreased by 50% with each facultative autogamous generation (recombination driven autoploidization). Regions of strict homology, where chiasma formation is likely, are more common in homologous than in homoeologous regions. Hence, recombination probabilities should increase in subsequent autogamy formed generations. Fortuitous loss or silencing of apomixis-causing alleles is likely in some lineages. For reproduction in these lineages to continue, reversion to sexual reproduction must occur. This may happen gradually or rapidly, with plants regaining complete sexual fertility after several generations of recombination.

To evaluate possibilities of apomixis-to-sex reversions in allodiploid *Boechera*, we searched the BMW for sexual endemics with limited geographic distributions and limited allelic variable (listed at the bottom of Supplementary Table S2). One of these, *B. mitchell-oldsiana*, is endemic to a 4 km stretch along the rim of Hells Canyon in northeastern Oregon. This location is within the center of diversity of two prominent sexual taxa, *B. retrofracta* and *B. sparsiflora*. Mean and median numbers of alleles per locus in the homozygous BMW samples for *B. mitchell-oldsiana* were 1.4 and 1, respectively (Supplementary Table S2). According to traditional views, *B. mitchell-oldsiana*, with its low allelic variability, could represent an ancient, nearly extinct sexual species that has experienced a genetic bottleneck followed by a weak comeback. Alternatively, it may have evolved from a single sexual species along an ecological gradient by directional selection. Then again, it may have evolved by reticulate evolution via a recombination-driven apomixis-to-sex reversion (Figure 4). If by directional selection, from a single species, most of its alleles should be found within single plants of its ancestral sexual species. However, if it evolved recently by apomixis-facilitated reticulation, its alleles should be found equally distributed between two ancestral sexual species, and local apomicts formed by hybridizations between these putative parental species should possess all or nearly all of the *B. mitchell-oldsiana* alleles.

As expected for an apomixis-facilitated reticulation, *B. mitchell-oldsiana* alleles were nearly evenly distributed between the two putative sexual parents, *B. retrofracta* and *B. sparsiflora*, and neither parent alone appeared to be capable of providing all of the needed alleles (Table 1, allele columns of putative sexual ancestors). In contrast, each of three local apomictic hybrids contained nearly all of the *B. mitchell-oldsiana* alleles (Table 1, allele columns for the three apomicts). The microsatellite-genotyped apomictic *B. retrofracta* × *sparsiflora* hybrids in the BMW represent only a small fraction of hundreds of such hybrids in this region from which *B. mitchell-oldsiana* may have evolved.

**Table 1:** Allele frequencies at 13 microsatellite loci for the allelically scant and geographically restricted sexual endemic *B. mitchell-oldsiana* (*mitc*) as found in the *Boechera* Microsatellite Website (BMW).

			Allele frequencies				Allele presence		
			*mitc* (38)	*retr* (72)	*spar* (24)	*pube* (19)	MS346	FW133	FW1042
Locus	Allele	Diploids with allele	OR	CA, ID, MI, MT, NV, OR, WA	CA, ID, NV, OR, WA	ID, NV, OR, UT, WA	CA	CA	OR
B11	80	35	1.00	1.00			×	×	×
A1	238	57	1.00	0.94	0.88	1.00	×	×	×
B18	115	31	0.03	0.71	0.46		×		×
114	217	11	1.00	0.63		0.05	×	×	×
B3	99	16	1.00	0.49	0.04			×	
B9	88	17	1.00	0.42	0.21		×	×	×
B15	93	18	1.00	0.33		0.11	×	×	
B20	207	23	1.00		0.50		×		
B18	113	23	0.97		0.46			×	
B6	301	17	1.00	0.26	0.33		×	×	×
B266	137	23	0.08		0.17		×		
13	81	26	0.37	0.04	0.13		×	×	
13	83	25	0.63		0.13	0.16	×		
B266	139	15	0.79	0.04	0.04		×	×	
B266	141	14	0.03	0.01	0.04				×
E9	202	15	1.00		0.04		×		×
B266	146	10	0.11	0.01					
C8	236	5	0.97			0.74			×
C8	256	17	0.03					×	
Sum: highest allele per locus	12.36	4.86	2.71	2.05	11	11	9
*mitc* loci represented (%)	100	69	77	38	85	85	69
Alleles not shown	0	72	64	38	14	19	21

*Corresponding values are presented for its three most allelically similar sexual taxa (putative progenitors), B. retrofracta (retr), B. sparsiflora (spar), and B. puberula (pube), and for three allelically similar triploid apomictic hybrids, the parents of which include one or more of the putative ancestors (MS346, B. retrofracta ×sparsiflora ×sparsiflora; FW133, B. rectissima ×retrofracta × sparsiflora; FW1042, B. cusickii ×puberula ×retrofracta). Numbers in the heading next to sexual taxa indicate number of homozygous lines in the BMW that were tallied to obtain frequency data. High frequency alleles from the two putative parents are shaded. For the three apomicts, allele presence values are from single plants. This highlights the possibility that sexual B. mitchell-oldsiana may have evolved from a single interspecific apomictic hybrid. Numbers of additional diploids that contain each allele are also listed (a measure of allele rarity). Locations are those from which BMW DNA samples were obtained. CAN, Canada; United States abbreviations as commonly used.*

## Discussion

### Evolutionary Instability: A Hallmark of Youthful *Boechera*

Angiospermous apomicts are typically perennial outcrossing polyploids that produce 1*n* pollen and, by a single apomixis type (e.g., apospory or diplospory), produce 2*n* female gametophytes and parthenogenetically competent eggs (Asker and Jerling, 1992; Carman, 1997, 2007). Apospory and diplospory occurring in the same plant is unusual. Where this occurs, the less frequent type is generally rarely observed, e.g., in *Tripsacum* and *Antennaria* (Carman, 2007), *Paspalum* (Bonilla and Quarin, 1997), *Rubus* (Czapik, 1996), *Poa* (Tian et al., 2013), and a few others (Nogler, 1984; Asker and Jerling, 1992; Carman, 1997). However, in several *Boechera* hybrids, apospory and diplospory occur simultaneously, each at elevated frequencies (Figure 1A). *Boechera* apomicts are atypical in other respects as well. For example, they are usually autogamous, instead of outcrossing, many produce 2*n* pollen, and many are diploid.

Figure 1A places taxa of like reproductive mode together. However, a closer look suggests a possible evolutionary relevance to this clustering. Specifically, the diplosporous apomicts (Figure 1A, 1–21) produce dyads of genetically unreduced spores in both male and female organs. In contrast, the aposporous apomicts (Figure 1A, 15–41) produce tetrads of genetically reduced spores in both female and male organs. Some of the aposporous apomicts are interracial hybrids, where fertile reduced pollen is expected (Figure 1A, 22–23, 33, 60). However, the others are allodiploids (Figure 1A, 24, 26–32, 35), which like diplosporous hybrids, should be sexually sterile or semisterile. Herein we propose a mechanism whereby new sexually fertile species may evolve from sexually semisterile but apomictically fertile allodiploids through facultative episodes of genome diploidization (Figure 4).

To reacquire meiotic stability after interspecific hybridization, apomictic *Boechera* must have undergone genome modifications that enhance chromosome pairing and recombination (diploidization). Since progeny of near-obligate apomicts are usually clonal and genetically identical to their mothers, well established allodiploid *Boechera* apomicts, which are also facultatively sexual, should have ample time (even hundreds of years) to sooner or later simultaneously produce 1*n* (genomically recombined) male and female gametes. In contrast, non-apomictic species hybrids are generally sterile, and these usually die without reproducing (Dobzhansky et al., 1977).

When allodiploid apomicts facultatively produce progeny by production and union of genomically recombined 1*n* gametes, a 50% reduction in homoeologous chromosome regions occurs. This is accompanied by a compensating increase in homologous (and homozygous) chromosome regions (Figure 4). With each additional autogamous generation, a 50% decrease in remaining homoeologous regions occurs. After several generations of selfing, each interspersed with perhaps multiple generations of apomixis, allodiploid apomicts should become sufficiently diploidized (Figure 4) for successful and efficient meioses to occur. Their chromosomes at this point represent chiasma-generated composites of alternating homozygous sections of the homoeologous genomes of their parents (Sybenga, 1996; Carman, 2007). This process is analogous to recombinant inbred line (RIL) production where multiple generations of selfing produce new chromosomes consisting of alternating segments of the original parental chromosomes. Chromosome painting studies provide evidence that such inter-genomic recombination in apomictic *Boechera* is extensive (Kantama et al., 2007; Koch, 2015).

If recombinational loss of parental chromosome regions eliminates alleles responsible for one apomixis type over another, or for apomixis in general, then new apomictic or sexual plants with uniquely recombined genomes may evolve (Figure 4). Such processes could explain the existence of a *B. imnahaensis* ×*yellowstonensis* accession that is mostly diplosporous and another accession of the same combination that is mostly aposporous (Figure 1A, 19, 27). It is noteworthy that many aposporous hybrids contain a *B. microphylla* clade genome (*B. thompsonii*, *B. imnahaensis*, or *B. yellowstonensis*), which suggests that the *B. microphylla* clade may be predisposed to switch from diplospory to apospory. Our data also suggest that tendencies toward apospory may persist for many sexual generations (Figure 1A, note high frequencies of tetrad formation with widely varying frequencies of apospory). While unreduced pollen is occasionally observed among apomicts of other angiospermous families, as well as among sexual plants (Asker and Jerling, 1992; Carman, 1997), the correlations between diplospory or apospory and 2*n* or 1*n* pollen, respectively, are unique to *Boechera*, and these correlations add to the uniqueness of the *Boechera* agamic complex.

The cytogenetic data available for the plants investigated herein (e.g., production of fertile 1*n* pollen) support the hypothesis of gradual, reticulation-driven shifts from recently evolved (sexually sterile or semisterile) diplosporous apomicts (Figure 1A, 1–14), to plants that produce 1*n* and 2*n* pollen and exhibit diplospory, apospory and sex (Figure 1A, 15–21), to plants that produce mostly 1*n* pollen and exhibit mostly apospory and sex (Figure 1A, 22–41), and finally to completely sexual plants that produce 1*n* pollen (Figure 1A, 42–64,B). It should be noted that only a very small percentage of progeny, if any, in each hybrid combination might fortuitously undertake this evolutionary route. In this respect, the vast majority of seeds produced by apomictic hybrids are genetic clones of the mother plant. Hence, while apomixis to sex reversions may on occasion occur for a given hybrid combination, the parental apomictic hybrid remains happily apomictic. The definitive test for verifying this process would be to observe it firsthand. As noted above, both diplosporous *B. exilis* ×*thompsonii* and aposporous *B. imnahaensis* × *yellowstonensis* produce about 4% of their seeds sexually. Accordingly, sexual gametophyte formation frequencies among sexually produced progeny (off types) could be determined. If segmental diploidization (Figure 4) and apomixis-to-sex reversions occur, they should be detectable within 2–4 generations. Another approach would be to genotype rare sexual endemics and their sexual and apomictic neighbors using phylogenetically stable markers. If a rare sexual endemic evolved recently from another sexual plant, most of its genetic markers should be similar to its progenitor. However, if it evolved by a recombination driven apomixis-to-sex reversal, then most or perhaps all of its molecular markers should be found in neighboring apomictic hybrids. In turn, these hybrids should contain near equal numbers of alleles from two distinct sexual parents, as was observed for *B. mitchell-oldsiana* herein (Table 1). Interestingly, *B. mitchell-oldsiana* exhibits a low frequency of apospory (Figure 1A, 41), which is consistent with a putative apomixis-to-sex origin.

The *B. mitchell-oldsiana* germplasm analyzed here is unique among SSR genotyped diploids. Specifically, it’s geographic distribution is restricted to a few flourishing populations, within 4 km of each other, in a single Oregon county. Similarly restricted populations of diploids have been reported, but only a few samples of SSR genotypes are available for them. It will be interesting to conduct analyses similar to that shown in Table 1 as additional rare sexual diploids are more thoroughly SSR genotyped.

Given the documented diversity of apomictic hybrids in *Boechera* [over 400 unique genomic combinations reported by Li et al. (2017)], it is evident that the association between apomixis and hybridization in this youthful genus is strong and that apomixis arises quickly following the amalgamation of divergent, mostly self-pollinating lineages. Likewise, if reversions from apomixis to sex require only a few successful sexual generations (Figure 4), then the entire process could reasonably occur in nature within a few decades. This would include (i) hybridization of sexual diploids, (ii) an homoeologous hybrid apomixis phase, (iii) a weakly apomictic segmental allodiploid phase, and (iv) a fully diploidized fledgling sexual endemic phase with early interspecific hybridizations of its own (Figure 1B). Variably repetitive patterns of microsatellite markers, as observed in the BMW (Li et al., 2017), could be explained by such a rapid recombination-driven speciation.

### Apomixis Types May Simply ReflectTemporal and Spatial Variations inTermination of Sexual Development andOnset of Gametophyte Formation

Apomixis in plants (Asker and Jerling, 1992), animals (Suomalainen et al., 1987), and protists (Bilinski et al., 1989) involves three single-cell processes: termination of sexual development, production of unreduced spores or eggs, and parthenogenesis where spores or eggs reinitiate the life cycle without syngamy. It has been hypothesized that these seminal events of apomixis are anciently polyphenic with the corresponding seminal events of sexual reproduction, and that eukaryotes in general have more or less retained, during evolution, abilities to switch from one reproductive mode (phenism) to the other (Carman et al., 2011; Hojsgaard et al., 2014; Albertini et al., 2019). Accordingly, onset timings and locations of unreduced gamete formation, which in angiosperms requires gametophyte formation, could be the event that defines apomixis types in angiosperms (Battaglia, 1989; Carman, 1997). If this hypothesis is correct, then apomixis types are not dependent on apomixis-type-specific mutations *per se* but on genetically controlled temporal and spatial variations in the induction of unreduced gametophyte formation.

Drought and heat stress can switch facultatively diplosporous *Boechera* from mostly apomeiotic dyad formation to mostly meiotic tetrad formation (Mateo de Arias, 2015). Hence, some of the variability in dyad to tetrad ratios observed among diplosporous accessions (Figure 1A, 1–21), especially those fixed in the field (Supplementary Table S1, Windham collections), may have been caused by variations in the weather prior to field collections.

**FIGURE 5:**
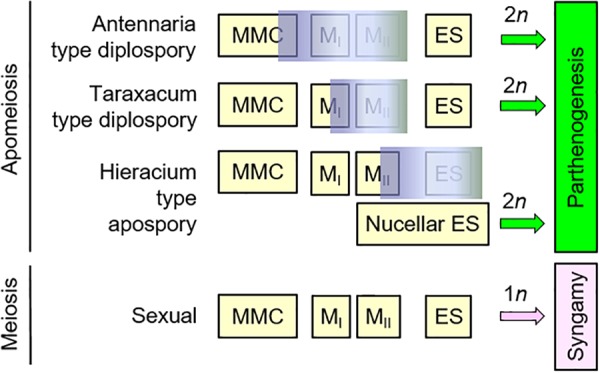
Timing of sexual program termination may determine apomixis type in *Boechera*. In Antennaria-type diplospory, the sexual program aborts early, meiosis fails completely, and the gametophyte (ES) forms directly from the megaspore mother cell (MMC). In Taraxacum-type diplospory, the sexual program aborts during meiotic prophase I. Restitution of the first meiotic division (the reductional division) then occurs, two unreduced spores form, and the ES generally forms from the chalazal most spore. In Hieracium-type apospory, sexual reproduction can be terminated as early as early meiosis or as late as early ES formation, with unreduced ESs forming adventitiously from sporogenous nucellar cells.

In certain ovules of the present study, sexual development was terminated prior to meiosis and was immediately replaced by unreduced gametophyte formation. This Antennaria-type diplospory occurred while integuments were still budding (Figure 2L,M). Likewise, unreduced gametophyte formation also followed the termination of sexual development during early meiotic prophase, which defines Taraxacum-type diplospory (Figure 2D,E), and shortly after meiosis in aposporous *Boechera* (Figure 2H,J). That multiple types of apomixis occur in the same plant is evidence that timing and location of unreduced gametophyte formation dictates apomixis type (Figure 5). Interestingly, high frequency shifts between types of apomixis, as well as between sexual and apomictic development, have been induced in sexual and apomictic *Boechera* through pharmacological treatments that affect stress response pathways and DNA methylation (Gao, 2018).

### Apomixis and Speciation, a Reappraisal

Facilitating the origins of genomically unique sexual species and genera runs counter to long held opinions concerning the involvement of apomixis in evolution. Historically, biologists considered apomixis, as well as wide hybridization and polyploidy, as antitheses of speciation (Darlington, 1939; Stebbins, 1971; Van Dijk and Vijverberg, 2005). Clearly, these processes block the selection-based shifts in allele frequencies thought to be required for gradual speciation along ecological gradients (Mayrose et al., 2011). However, studies now implicate reticulation as a prominent player in speciation (Carman, 1997; Rieseberg, 1997; Martis et al., 2013; Sochor et al., 2015; Payseur and Rieseberg, 2016; Vargas et al., 2017; Hojsgaard, 2018). In this respect, the immortality conferred by apomixis to allodiploid *Boechera* should provide them with unlimited time for rare recombinations to occur and for sexually fertile species, which possess multi-species-recombinant genomes, to evolve (Figure 4). To date, only a few cases of apomixis to sex reversions have been reported (Chapman et al., 2003; Domes et al., 2007; Horandl and Hojsgaard, 2012; Ortiz et al., 2013; Hojsgaard et al., 2014). However, this could change if the speciation mechanism described herein is found to be of more general occurrence among agamic complexes.

Establishment of apomixis-to-sex founder plants, like the recombinational events required to generate them, are probably rare, and this rarity could explain the low levels of allelic variability observed among some of the sexual diploids of *Boechera* (Supplementary Table S2). Also, since geographic ranges of apomicts often exceed those of their sexual progenitors (Bayer, 1997; Hojsgaard et al., 2014), newly formed apomixis-to-sex founder populations could reasonably be allopatric with their most recent sexual ancestors but sympatric with clones of their immediate apomictic parents.

Few diploid apomicts exist outside of *Boechera*. Generally, they are ephemeral, sexually sterile, and apomictically fertile allodiploids that occasionally form from allotetraploids through parthenogenesis of 1*n* (=2*x*) eggs (Asker and Jerling, 1992). Spontaneous haploid parthenogenesis is reasonably common in angiosperms (Dunwell, 2010). Hence, most allotetraploid apomicts probably on occasion produce allodiploids. Published examples have been reported in the following genera: *Parthenium* (Gerstel and Mishanec, 1950), *Hierochloe* (Weimarck, 1967), *Ranunculus* (Nogler, 1982), *Allium* (Kojima and Nagato, 1997), *Hieracium* (Bicknell, 1997), and *Erigeron* (Noyes and Wagner, 2014). If the genomes of such allodiploids are sufficiently divergent as to prevent the formation of fertile and reduced gametes, then apomixis, provided it is still occurring (Noyes and Wagner, 2014), could stabilize the cytotype. However, if the allodiploid apomict happens to produce progeny sexually, especially by selfing, then recombination driven diploidization with the formation of genomically unique sexual species may eventually occur (Figure 4).

## Conclusion

The unique situation in *Boechera* of self-pollinating, aposporously fertile allodiploids that facultatively produce 1*n* eggs and sperm may facilitate reversions from apomixis to sex. In fact, multiple genomically unique sexual species could in theory evolve from the same allodiploid, the divergent genomes of which would contain different assortments of homozygous segments of the original parental genomes (Figure 4). In this manner, apomixis may serve as an effective springboard in stabilizing reticulate evolution processes (Carman, 1997). Since allodiploidy increases rates of homoeologous recombination (Dewey, 1984; Wang, 1989; Grandont et al., 2014; Poggio et al., 2016), diploidization possibilities should be enhanced. Accordingly, the aposporous allodiploid *Boechera* identified herein are well suited for studying this putative speciation mechanism. While occurring at a much slower pace, this process could also occur among polyploid apomicts. Here, the process would originate in allodiploids that form from allotetraploid apomicts by haploid parthenogenesis.

## Author Contributions

JC designed the study and wrote the manuscript with important contributions from MW and DS. MMdA, MS, and KD conducted the flow cytometry. MW provided the taxonomic guidance. MW, JC, DS, LG, and MS collected the specimens. BK and JC designed the *Boechera* embryology procedures. JC, MMdA, XZ, LG, BK, DS, MS, BP, and LW conducted the embryological analyses. All authors read and approved the final draft.

## Conflict of Interest Statement

The authors declare that the research was conducted in the absence of any commercial or financial relationships that could be construed as a potential conflict of interest.

## Supplementary Material

FIGURE S1 Additional examples of meiotic tetrads and immature 1- to 2-nucleate gametophytes (ESs) forming from functional (surviving) megaspores (FMs) in sexual *Boechera*. Tetrads and distinctly vacuolate ESs forming from FMs are diagnostic of sexual reproduction. Numbers after taxa correspond to accession numbers in Figure 1 and Supplementary Table S1. **(A,B)** *B. formosa*, 52; **(C,D)** *B. schistacea*, 56; **(E,F)** *B. pendulina*, 61; **(G,H)** *B. stricta*, 62; **(I,J)** *B. lemmonii*, 64; **(K,L)** *B. oxylobula*, 59; **(M,N)** *B. juniperina*, 44; **(O,P)** *B. sparsiflora*, 58. Black arrows, degenerating megaspores; white arrows, surviving megaspores or early developing gametophytes; narrow white lines, central column of nucellar cells, which gave rise to the archesporial cell; P, parietal cell; bars, 20 μm.

FIGURE S2 Additional examples of diplosporous and aposporous reproduction in *Boechera*. Distinctly vacuolate gametophytes (ESs) that form from the chalazal member of a dyad are diagnostic of Taraxacum-type diplospory **(A–F)**. Distinctly vacuolate ESs that form from nucellar cells and replace degenerating tetrads are diagnostic of apospory **(H–O)**. Numbers after taxa correspond to accession numbers in Figure 1 and Supplementary Table S1. **(A)** partial row of unreduced Taraxacum-type diplosporous dyads in a *B. exilis* ×*thompsonii*, 9, pistil; **(B)** *B. exilis* ×*retrofracta*, 1; **(C)** *B. pendulina* ×*thompsonii*, 11; **(D)** *B. fendleri* ×*stricta*, 16; **(E)** *B. cf. gunnisoniana* 3×, 6; **(F)** *B. exilis* ×*retrofracta*, 2; **(G)** unreduced microspore dyads from a 1.1 mm long *B. retrofracta* × *stricta*, 21, anther. **(H)** *B. thompsonii* ×*thompsonii*, 33; **(I)** *B. thompsonii* × *thompsonii*, 22; **(J)** *B. crandallii* ×*thompsonii*, 31 (additional focal plane of Figure 2I); **(K)** two unreduced 1 nucleate Hieracium-type aposporous ESs, an aposporous initial, and a degenerating tetrad in a *B. retrofracta* ×*stricta* ovule, 21; **(L)** *B. cusickii* ×*sparsiflora*, 24; **(M)** *B. fendleri* ×*stricta*, 32; **(N)** unreduced 1 nucleate Hieracium-type aposporous ES with degenerating unreduced Taraxacum-type dyad in a *B. retrofracta* ×*stricta* ovule, 21; **(O)** *B. exilis* ×*thompsonii*, 30; black arrows, degenerating megaspores; white arrows, surviving megaspores; narrow white lines, central column of nucellar cells, which gave rise to the archesporial cell; AES1 and 2, 1- and 2-nucleate aposporous ESs, respectively; DES1 and 2, 1- and 2-nucleate diplosporous ESs, respectively; Dy, microspore dyads; P, parietal cell; bars, 20 μm.

TABLE S1 Collection information for *Boechera* accessions evaluated cytologically for mode of reproduction. Numbers following taxa names correspond to numbered taxa in Figure 1; BMW, *Boechera* Microsatellite Website; collection numbers are those of Carman (JC) and Windham (MW).

TABLE S2 Numbers of SSR alleles observed among the homozygous samples of 59 diploid sexual *Boechera* taxa as of July, 2017.
