# Supplementary figures and images for: Apospory and Diplospory in Diploid Boechera (Brassicaceae) May Facilitate Speciation by Recombination-Driven Apomixis-to-Sex Reversals
Source: Front Plant Sci. 2019 May 31;10:724. doi: 10.3389/fpls.2019.00724 (PMC6555261; doi:10.3389/fpls.2019.00724)

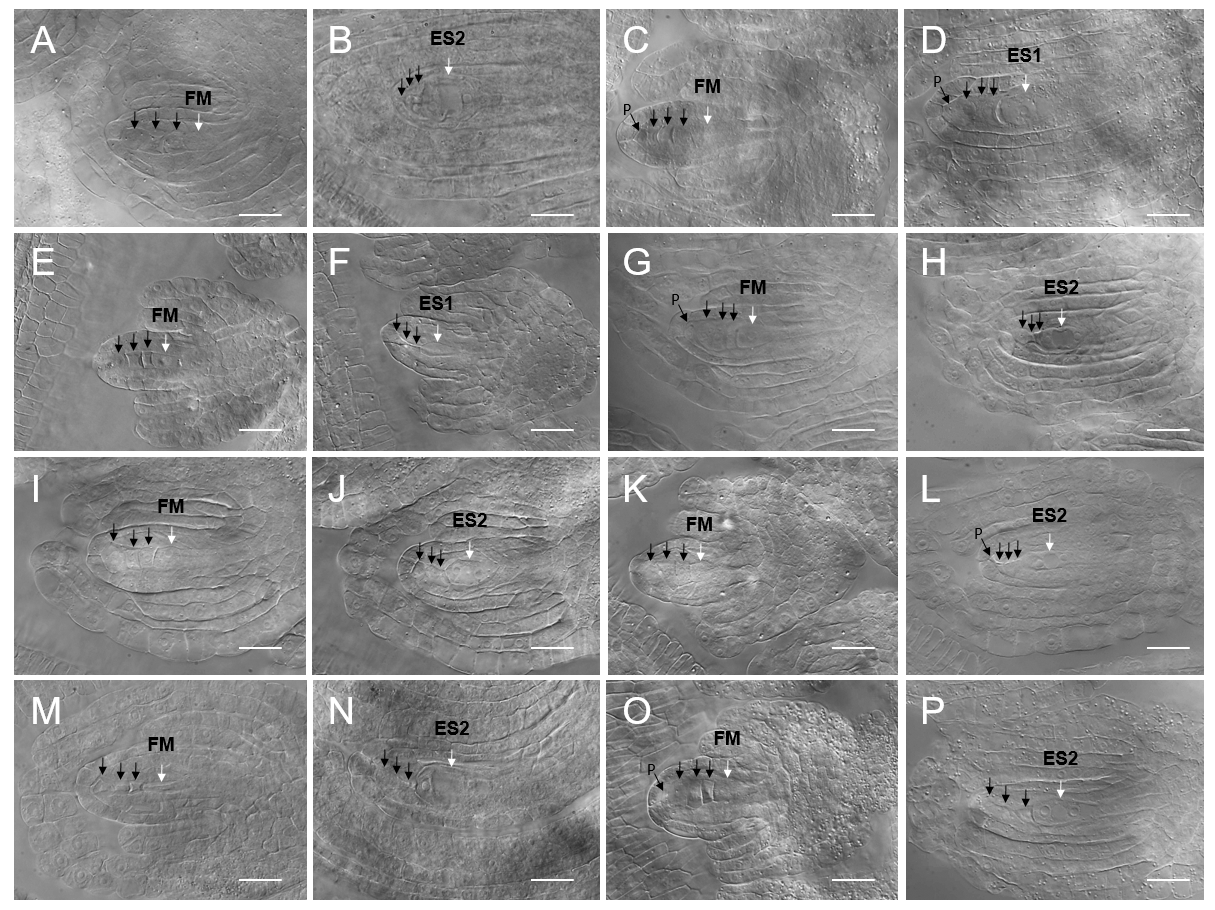

Supplement: FIGURE S1 — Additional examples of meiotic tetrads and immature 1- to 2-nucleate gametophytes (ESs) forming from functional (surviving) megaspores (FMs) in sexual Boechera. Tetrads and distinctly vacuolate ESs forming from FMs are diagnostic of sexual reproduction. Numbers after taxa correspond to accession numbers in Figure 1 and Supplementary Table S1. (A,B) B. formosa, 52; (C,D) B. schistacea, 56; (E,F) B. pendulina, 61; (G,H) B. stricta, 62; (I,J) B. lemmonii, 64; (K,L) B. oxylobula, 59; (M,N) B. juniperina, 44; (O,P) B. sparsiflora, 58. Black arrows, degenerating megaspores; white arrows, surviving megaspores or early developing gametophytes; narrow white lines, central column of nucellar cells, which gave rise to the archesporial cell; P, parietal cell; bars, 20 μm. [file Image_1.TIF]

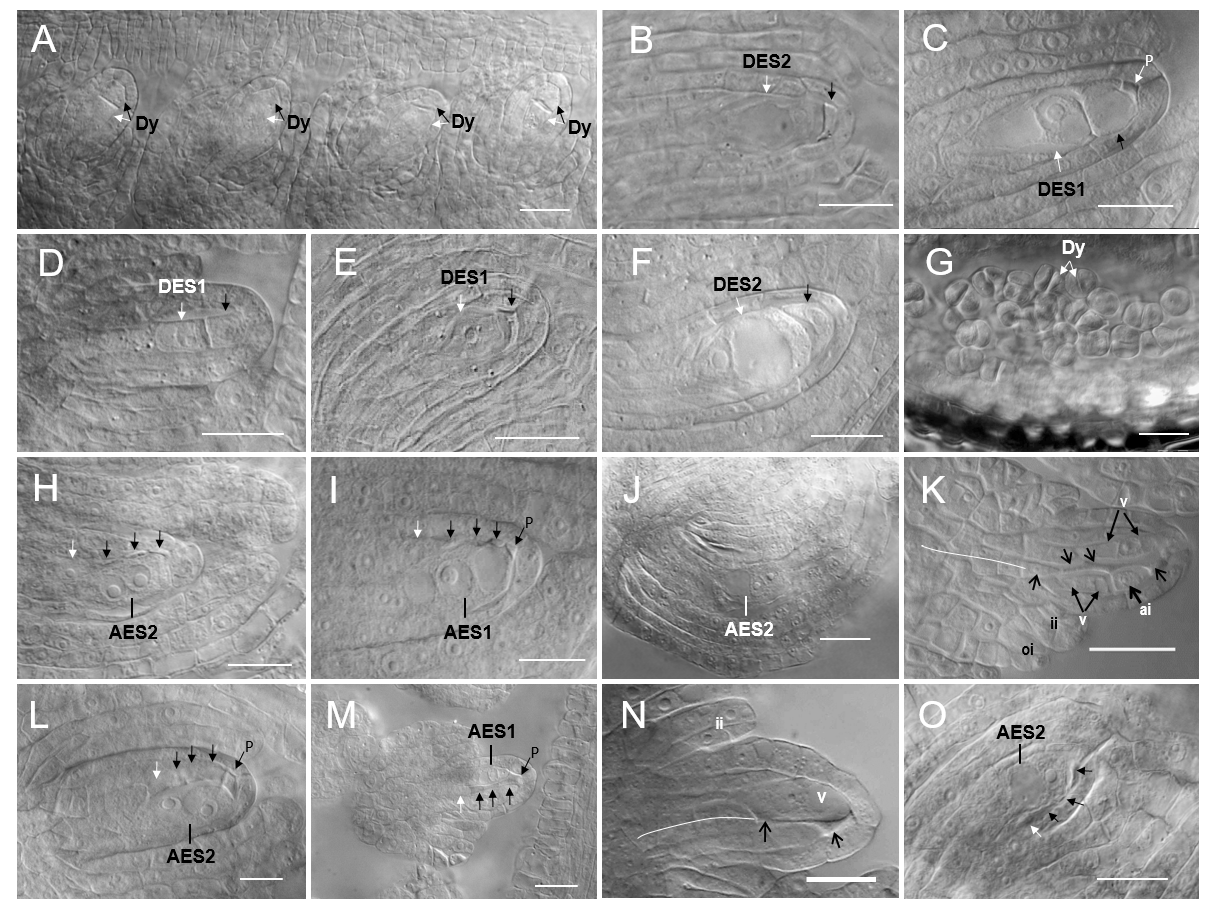

Supplement: FIGURE S2 — Additional examples of diplosporous and aposporous reproduction in Boechera. Distinctly vacuolate gametophytes (ESs) that form from the chalazal member of a dyad are diagnostic of Taraxacum-type diplospory (A–F). Distinctly vacuolate ESs that form from nucellar cells and replace degenerating tetrads are diagnostic of apospory (H–O). Numbers after taxa correspond to accession numbers in Figure 1 and Supplementary Table S1. (A) partial row of unreduced Taraxacum-type diplosporous dyads in a B. exilis ×thompsonii, 9, pistil; (B) B. exilis ×retrofracta, 1; (C) B. pendulina ×thompsonii, 11; (D) B. fendleri ×stricta, 16; (E) B. cf. gunnisoniana 3×, 6; (F) B. exilis ×retrofracta, 2; (G) unreduced microspore dyads from a 1.1 mm long B. retrofracta × stricta, 21, anther. (H) B. thompsonii ×thompsonii, 33; (I) B. thompsonii × thompsonii, 22; (J) B. crandallii ×thompsonii, 31 (additional focal plane of Figure 2I); (K) two unreduced 1 nucleate Hieracium-type aposporous ESs, an aposporous initial, and a degenerating tetrad in a B. retrofracta ×stricta ovule, 21; (L) B. cusickii ×sparsiflora, 24; (M) B. fendleri ×stricta, 32; (N) unreduced 1 nucleate Hieracium-type aposporous ES with degenerating unreduced Taraxacum-type dyad in a B. retrofracta ×stricta ovule, 21; (O) B. exilis ×thompsonii, 30; black arrows, degenerating megaspores; white arrows, surviving megaspores; narrow white lines, central column of nucellar cells, which gave rise to the archesporial cell; AES1 and 2, 1- and 2-nucleate aposporous ESs, respectively; DES1 and 2, 1- and 2-nucleate diplosporous ESs, respectively; Dy, microspore dyads; P, parietal cell; bars, 20 μm. [file Image_2.TIF]
