# Supplementary material for: Apospory and Diplospory in Diploid Boechera (Brassicaceae) May Facilitate Speciation by Recombination-Driven Apomixis-to-Sex Reversals
Source: Front Plant Sci. 2019 May 31;10:724. doi: 10.3389/fpls.2019.00724 (PMC6555261; doi:10.3389/fpls.2019.00724)
Supplement: TABLE S1 — Collection information for Boechera accessions evaluated cytologically for mode of reproduction. Numbers following taxa names correspond to numbered taxa in Figure 1; BMW, Boechera Microsatellite Website; collection numbers are those of Carman (JC) and Windham (MW). [file Table_1.DOCX]

| **Table S1.** Collection information for *Boechera* accessions evaluated cytologically for mode of reproduction. Numbers following taxa names correspond to numbered taxa in Fig. 1; BMW, *Boechera* Microsatellite Website; collection numbers are those of Carman (JC) and Windham (MW). | | | | |
| --- | --- | --- | --- | --- |
| *Boechera* accession | Collection & voucher location | Collection locality | Lat. Long. | BMW no. |
| *caeruleamontana* (39) | MW 4372, DUKE | UT, Uintah Co. | 40.3516 -109.1734 | LA341 |
| *crandallii* (48) | MW 4032, DUKE | CO, Gunnison Co. | 38.7506 -106.7680 | FW276 |
| *crandallii x gracilipes* (20) | MW 4207, NY | CO, Ouray Co. | 38.0654 -107.6713 | FW1572 |
| *crandallii x thompsonii* (31) | MW 4029, DUKE | CO, Montrose Co. | 38.4468 -107.5551 | FW274 |
| *cusickii x kelseyana x pendulina* (3) | MW 99-100, UT | UT, Box Elder Co. | 41.8659 -113.7823 | JB399 |
| *cusickii x sparsiflora* (24) | MW 99-224, DUKE | NV, Elko Co. | 41.3242 -115.9678 | JB102 |
| *exilis* (43) | JC UT10011, UTC | UT, Summit Co. | 40.7753 -111.4078 | CR1164 |
| *exilis* (44) | JC NV4003, UTC | NV, Elko Co. | 41.1150 -114.8141 | ---------- |
| *exilis* (45) | MW3987, DUKE | NV, Elko Co. | 41.1136 -114.8135 | FW242 |
| *exilis x retrofracta* (1) | JC UT11004, UTC | UT, Utah Co. | 40.1944 -111.3831 | JB754 |
| *exilis x retrofracta* (42) | MW 3691, DUKE | ID, Bingham Co. | 43.3058 -112.2714 | FW80 |
| *exilis x retrofracta* (2) | MW 4378, DUKE | UT, Wasatch Co. | 40.5934 -111.4160 | LA221 |
| *exilis x thompsonii* (8) | JC WY05001, UTC | WY, Sweetwater Co. | 41.5517 -109.5253 | CR1358 |
| *exilis x thompsonii* (9) | MW 3876, DUKE | UT, Uintah Co. | 40.2903 -109.1331 | JB1270 |
| *exilis x thompsonii* (30) | MW 3914, DUKE | NE, Eureka Co. | 39.5727 -116.0772 | JB1303 |
| *fendleri x stricta* (32) | MW 4182, DUKE | CO, Archuleta Co. | 37.1078 -106.9145 | FW1543 |
| *fendleri x stricta* (16) | MW 3600, DUKE | UT, Kane Co. | 37.5080 -112.6217 | JB562 |
| *fernaldiana* (51) | MW 3910, DUKE | NV, Churchill Co. | 39.5736 -117.8560 | JB1299 |
| *formosa* (52) | MW 99-051, UT | UT, Grand Co. | 38.7042 -109.3869 | JB1407 |
| *formosa* (53) | JC UT10006, UTC | UT, Duchesne Co. | 40.1692 -110.3279 | FW1162 |
| *formosa x thompsonii* (17) | JC UT05002, UTC | UT, Duchesne Co. | 40.1692 -110.3279 | FW1161 |
| *formosa x thompsonii* (28) | MW 3003, DUKE | UT, Duchesne Co. | 40.1695 -110.3281 | JB209 |
| *gracilipes* (38) | MW 3593, DUKE | AZ, Coconino Co. | 35.2036 -111.6414 | JB124 |
| *gracilipes x kelseyana x perennans* (7) | MW 2319, DUKE | AZ, Apache Co. | 35.8753 -109.4432 | JB427 |
| *gracilipes x perennans* (26) | MW 3578a, DUKE | UT, Beaver Co. | 38.2545 -112.5291 | JB225 |
| *gracilipes x perennans* (35) | MW 3083, DUKE | UT, Millard Co. | 39.3497 -112.2658 | ---------- |
| *gracilipes x retrofracta* (4) | MW 3382, DUKE | CO, Archuleta Co. | 37.2871 -107.0822 | JB299 |
| *gracilipes x retrofracta* (13) | MW 4023, DUKE | CO, Ouray Co. | 38.0189 -107.6831 | FW270 |
| *gracilipes x thompsonii* (5) | MW 3989, DUKE | UT, Beaver Co. | 38.3741 -112.8295 | FW244 |
| *gunnisoniana x ?* 3x (6) | JC CO11005, UTC | CO, Gunnison Co. | 38.5274 -106.8144 | ---------- |
| *imnahaensis* (57) | JC OR12002, UTC | OR, Wallowa Co. | 45.8160 -116.7654 | FW1158 |
| *imnahaensis x yellowstonensis* (19) | JC UT05001, UTC | UT, Cache County | 41.7952 -111.6470 | ---------- |
| *imnahaensis x yellowstonensis* (27) | JC UT10003, UTC | UT, Millard Co. | 39.3500 -112.2642 | CR1324 |
| *juniperina* (44) | MW 3425, DUKE | CA, San Bernardino Co. | 34.2722 -116.7806 | JB107 |
| *kelseyana* (49) | MW 3950a, DUKE | NM, Sandoval Co. | 36.2083 -107.5629 | FW228 |
| *laevigata* (34) | MW,4087, DUKE | MI, Shiawassee Co. | 43.0862 -84.1838 | FW1134 |
| *laevigata* (37) | MW 3449, DUKE | MO, McDonald Co. | 36.6317 -94.5903 | JB234 |
| *laevigata x stricta* (2:2) (40) | MW 4089, DUKE | MI, Alcona Co. | 44.5236 -83.6449 | FW1111 |
| *lemmonii* (64) | MW 2483, DUKE | CA, Mono Co. | 38.4400 -119.3242 | JB318 |
| *lemmonii x mitchell-oldsiana*  (29) | MW 4130, DUKE | OR, Wallowa Co. | 45.2348 -117.0833 | FW1246 |
| *mitchell-oldsiana* (41) | MW 4114, DUKE | OR, Wallowa Co. | 45.4018 -116.7226 | FW1188 |
| *mitchell-oldsiana x* retrofracta 1:1 (18) | MW 4119, DUKE | OR, Wallowa Co. | 45.4046 -116.7219 | FW1224 |
| *mitchell-oldsiana x retrofracta* 2:1 (10) | MW 4126, DUKE | OR, Wallowa Co. | 45.4632 -116.6544 | FW1238 |
| *mitchell-oldsiana x retrofracta* 2:1 (14) | MW 4115, DUKE | OR, Wallowa Co. | 45.4018 -116.7226 | FW1211 |
| *mitchell-oldsiana x retrofracta* 2:1 (15) | MW et al. 4117 DUKE | OR, Wallowa Co. | 45.4031 -116.7222 | FW1220 |
| *oxylobula* (59) | MW 3052, DUKE | CO, Montrose Co. | 38.4556 -107.5456 | JB141 |
| *pendulina* (61) | MW 2025, NY | UT, Kane Co. | 37.4912 -112.5631 | FW656 |
| *pendulina x thompsonii* (11) | MW 3364, DUKE | UT, Carbon Co. | 39.5471 -110.6483 | JB338 |
| *pendulina x wyomingensis* (12) | MW 2078, DUKE | WY, Sweetwater Co. | 41.0678 -109.2792 | JB255 |
| *puberula* (54) | JC NV10004 | NV, Elko Co. | 40.2570 -114.2654 | -------- |
| *puberula* (55) | JC NV10003 | NV, Elko Co. | 40.2592 -114.2649 | -------- |
| *pulchra* (50) | MW 3435, DUKE | CA, Kern Co. | 35.5808 -118.4244 | JB270 |
| *retrofracta x retrofracta* (25) | MW 4103, DUKE | ID, Adams Co. | 44.6428 -116.2362 | FW1176 |
| *retrofracta x stricta* (21) | JC CO11010, UTC | CO, Rio Blanco Co. | 39.7029 -107.9986 | PJA374a (P. Alexander) |
| *schistacea* (56) | MW 99-284, UT | NV, Nye Co. | 38.9523 -117.3380 | JB365 |
| *sparsiflora* (58) | MW 3647, DUKE | OR, Malheur Co. | 44.2275 -117.0500 | JB280 |
| *stricta* (62) | MW 2128, DUKE | UT, Piute Co. | 38.29 -112.0622 | JB1124 |
| *stricta* (63) | JC UT10007, UTC | UT, Duchesne Co. | 40.5559 -110.8918 | --------- |
| *thompsonii* x *thompsonii* (33) | MW 3080, DUKE | UT, San Juan Co. | 37.9181 -109.9453 | JB335 |
| *thompsonii* x *thompsonii* (36) | MW 2313,NY | UT, San Juan, Co. | 38.3051 -109.3625 | FW948 |
| *thompsonii* x *thompsonii* (22) | MW 3923 | UT, Duchesne Co. | 40.1708 -110.3318 | FW199 |
| *thompsonii* x *thompsonii* (23) | MW 3931, DUKE | UT, San Juan Co. | 37.9759 -109.8462 | FW208 |
| *thompsonii* (60) | MW 3925, DUKE | UT, Carbon Co. | 39.6441 -110.6307 | FW201 |
| *yellowstonensis* (47) | JC WY12003, UTC | WY, Park Co. | 44.4559 -109.5285 | FW1159 |
