# Supplementary material for: Apospory and Diplospory in Diploid Boechera (Brassicaceae) May Facilitate Speciation by Recombination-Driven Apomixis-to-Sex Reversals
Source: Front Plant Sci. 2019 May 31;10:724. doi: 10.3389/fpls.2019.00724 (PMC6555261; doi:10.3389/fpls.2019.00724)
Supplement: TABLE S2 — Numbers of SSR alleles observed among the homozygous samples of 59 diploid sexual Boechera taxa as of July, 2017. [file Table_2.DOCX]

| **Table S2.** Numbers of SSR alleles observed among the homozygous samples of 59 diploid sexual *Boechera* taxa. | | | | | |
| --- | --- | --- | --- | --- | --- |
| Alleles per locus | | Taxon | No. | Country collected | States (with number of counties) or provinces where collections were made |
| Median | Mean |  |  |  |  |
| Median number of alleles per locus in the observed populations of homozygotes: 5-10 | | | | | |
| 10.0 | 8.9 | *stricta* | 101 | CAN, USA | AB; BC; NT; SK; YT; AL, 1; AZ, 1; CA, 5; CO, 17; ID, 3; MT, 3; NV, 3; NM, 2; OR, 1; SD, 1; UT, 13; WA, 4; WY, 7 |
| 11.0 | 9.5 | *lemmonii* | 59 | CAN, USA | AB; BC; CA, 6; CO, 6; ID, 4; MT, 4; NV, 6; OR, 5; UT, 5; WA, 1; WY, 4 |
| 6.0 | 6.2 | *retrofracta* | 69 | CAN, USA | AL; BC; NT; ON; QC; SK; YT; AK; CA, 4; ID, 5; MI, 1; MT, 2; NV, 1; OR, 4; WA, 11 |
| 5.0 | 6.2 | *thompsonii* | 42 | USA | AZ, 1; CO, 7; NM, 5; UT, 5; WY, 1 |
| 6.0 | 5.2 | *laevigata* | 18 | USA | CO, 1; IL, 1; IN, 2; IA, 1; KS, 1; MO, 1; NY, 1; NC, 1; OH, 1; PA, 2; TN, 2; VA, 1; WV, 3 |
| 6.0 | 8.5 | *perennans* | 76 | USA | AZ, 8; CA, 4; NV, 1; NM, 4; UT, 1 |
| 9.0 | 7.2 | *pendulina* | 35 | USA | AZ, 1; CA, 1; NV, 5; UT, 10 |
| 7.0 | 6.5 | *fendleri* | 42 | USA | CO, 1; NM, 12; UT, 2 |
| 5.0 | 5.3 | *subpinnatifida* | 16 | USA | CA, 4; OR, 2 |
| Median number of alleles per locus in the observed populations of homozygotes: 4-4.5 | | | | | |
| 4.0 | 5.8 | *sparsiflora* | 24 | USA | CA, 5; ID, 5; NV, 2; OR, 4; WA, 3 |
| 4.0 | 4.8 | *pendulocarpa* | 22 | CAN, USA | AL; BC; SK; YT; CA, 2; ID, 1; MT, 1; NV, 2; WY, 3 |
| 4.5 | 4.9 | *oxylobula* | 26 | USA | CO, 10 |
| 4.0 | 4.2 | *davidsonii* | 15 | USA | CA, 8; NV, 1 |
| 4.0 | 5.0 | *wyomingensis* | 30 | USA | CO, 1; UT, 1; WY, 6 |
| 4.0 | 3.6 | *imnahaensis* | 18 | USA | ID, 1; OR, 4; WA, 2 |
| Median number of alleles per locus in the observed populations of homozygotes: 3-3.5 | | | | | |
| 3.0 | 3.8 | *nubigena* | 28 | CAN, USA | AL; BC; ID, 7; MT, 1; OR, 6; WA, 3; WY, 1 |
| 3.0 | 3.3 | *cobrensis* | 22 | USA | CA, 1; ID, 2; NV, 11; OR, 3; UT, 1 |
| 3.0 | 5.0 | *cusickii* | 29 | USA | ID, 3;NV, 1; OR, 7; WA, 7 |
| 3.0 | 3.5 | *formosa* | 21 | USA | AZ, 1; CO, 3; NM, 2; UT, 6; WY, 1 |
| 3.5 | 4.3 | *arida* | 20 | USA | CA, 2; NV, 9; OR, 2 |
| 3.0 | 3.7 | *gracilipes* | 35 | USA | AZ, 4; NV, 2; NM, 1; UT, 4 |
| 3.0 | 3.8 | *puberula* | 18 | USA | ID, 3; NV, 2; OR, 4; UT, 1; WA, 1 |
| 3.0 | 4.1 | *shockleyi* | 41 | USA | CA, 3; NV, 5; UT, 3 |
| 3.0 | 3.8 | *arcuata* | 16 | USA | CA, 10 |
| 3.0 | 4.2 | *schistacea* | 63 | USA | NV, 3; UT, 4; WY, 1 |
| 3.0 | 4.2 | *hoffmannii* | 26 | USA | CA, 1; NV, 5 |
| 3.0 | 3.2 | *ursalaca* | 15 | USA | ID, 1; WY, 3 |
| 3.0 | 3.2 | *fecunda* | 18 | USA | MT, 3 |
| Median number of alleles per locus in the observed populations of homozygotes: 2-2.5 | | | | | |
| 2.0 | 3.2 | *spatifolia* | 46 | USA | CO, 15; WY, 2 |
| 2.0 | 3.0 | *rectissima* | 20 | USA | CA, 13; OR, 1 |
| 2.0 | 3.1 | *pulchra* | 26 | USA | CA, 8; NV, 4 |
| 2.0 | 3.2 | *howellii* | 17 | USA | CA, 10; OR, 1 |
| 2.0 | 3.2 | *paupercula* | 20 | USA | CA, 7; OR, 3 |
| 2.0 | 3.1 | *breweri* | 11 | USA | CA, 10 |
| 2.0 | 2.7 | *lincolnensis* | 36 | USA | CA, 1; NV, 6; UT, 2 |
| 2.0 | 3.1 | *platysperma* | 8 | USA | CA, 8 |
| 2.0 | 3.0 | *murrayi* | 14 | CAN, USA | YT; CA, 2; ID, 1; MT, 1; WY, 2 |
| 2.0 | 2.9 | *glaucovalvula* | 23 | USA | CA, 5; NV, 2 |
| 2.0 | 2.4 | *kelseyana* | 18 | USA | AZ, 2; NM, 3 |
| 2.0 | 3.6 | *texana* | 20 | USA | TX, 5 |
| 2.0 | 2.2 | *uintaensis* | 14 | USA | WY, 5 |
| 2.0 | 2.8 | *nevadensis* | 44 | USA | CA, 1; NV, 3 |
| 2.5 | 3.2 | *fernaldiana* | 8 | USA | CA, 1; NV, 3 |
| 2.0 | 2.3 | *atrorubens* | 18 | USA | OR, 1; WA, 3 |
| 2.0 | 2.1 | *serpenticola* | 10 | USA | CA, 3 |
| 2.0 | 3.2 | *shastaensis* | 12 | USA | CA, 2 |
| 2.0 | 2.6 | *pygmaea* | 8 | USA | CA, 2 |
| 2.0 | 2.5 | *williamsii* | 15 | USA | WY, 2 |
| Median number of alleles per locus in the observed populations of homozygotes: 1-1.5 | | | | | |
| 1.0 | 1.3 | *exilis* | 24 | USA | CO, 1; NV, 3; UT, 5; WY, 5 |
| 1.0 | 1.8 | *saskatchewanensis* | 19 | CAN, USA | AL; MB; SK; MT, 1; NB, 1; ND, 1; SD,2; WY, 1 |
| 1.5 | 1.9 | *crandallii* | 16 | USA | CO, 7 |
| 1.5 | 4.2 | *lasiocarpa* | 24 | USA | UT, 6 |
| 1.5 | 2.3 | *stipitata* | 9 | USA | CA, 2; OR, 3 |
| 1.0 | 2.5 | *juniperina* | 13 | USA | CA, 3; NV, 1 |
| 1.0 | 1.8 | *dispar* | 10 | USA | CA, 2; NV, 1 |
| 1.0 | 1.4 | *mitchell-oldsiana* | 38 | USA | OR, 1 |
| 1.0 | 1.4 | *koehleri* | 8 | USA | OR, 1 |
| 1.0 | 1.3 | *yellowstonensis* | 7 | USA | WY, 1 |
| 1.0 | 1.2 | *evadens* | 7 | USA | CA, 1 |
| Only taxa that were homozygous for the 13 single-locus microsatellite loci in the *Boechera* Microsatellite Website as of June 21, 2017 are shown; Canadian and U.S. abbreviations as commonly used. | | | | | |
